# Prenatal Evidence of Persistent Notochord and Absent Sacrum Caused by a Mutation in the T (Brachyury) Gene

**DOI:** 10.1155/2016/7625341

**Published:** 2016-12-26

**Authors:** F. Fontanella, M. C. van Maarle, P. Robles de Medina, R. J. Oostra, R. R. van Rijn, E. Pajkrt, C. M. Bilardo

**Affiliations:** ^1^Department of Obstetrics, Gynaecology and Prenatal Diagnosis, University Medical Center Groningen, University of Groningen, Groningen, Netherlands; ^2^Department of Clinical Genetics, Academic Medical Center Amsterdam, Amsterdam, Netherlands; ^3^Department of Obstetrics and Gynaecology, Academic Medical Center Amsterdam, Amsterdam, Netherlands; ^4^Department of Anatomy and Embryology, Academic Medical Center Amsterdam, Amsterdam, Netherlands; ^5^Department of Radiology, Academic Medical Center Amsterdam, Amsterdam, Netherlands

## Abstract

Caudal regression syndrome (CRS) is a rare congenital disorder characterized by developmental abnormalities of caudal spinal segments. To date, the etiology of CRS is unclear; sporadic cases are strongly associated with maternal diabetes, while familiar recurrence is infrequent. We describe in detail the prenatal clinical and sonographic findings of a recently described hereditary caudal regression syndrome, in four fetuses reported to be homozygous for a mutation in the T (brachyury) gene. The syndrome occurred in three consanguineous, but unrelated families, originating from the same geographical area. All affected fetuses had persistence of the notochord in association with abnormal vertebral ossification, sacral agenesis, and bilateral clubfoot. These findings suggest that, in case of prenatal diagnosis of sacral agenesis, an advanced ultrasound examination should assess the vertebral ossification and the rare persistence of the notochord, in order to rule the involvement of the T gene.

## 1. Introduction

Sacral agenesis (caudal regression syndrome or CRS) is a rare congenital syndrome characterized by developmental abnormalities of caudal spinal segments and by a wide spectrum of phenotypes, ranging from minor sacrococcygeal malformations to complete absence of sacrum and lumbar spine. Other associated urogenital, gastrointestinal, or cardiac pathologies may occur [1].

Poorly controlled diabetes is considered as the main risk factor besides possible teratogenic causes [2]. However, cases of CRS have been also reported in absence of drug exposure or maternal diabetes [3]. Genetic factors have been proposed, but the absence of recurrence of identical malformations in subsequent pregnancies did not support this hypothesis [4].

Up to now, a causative gene (MNX1) has been found only in patients with the Currarino syndrome, also known as hereditary sacral agenesis and characterized by sacral defect, anorectal malformation, and presacral mass [5].

## 2. Case Presentation

### 2.1. Family A: Case 1

A 29-year-old woman, gravida 3 para 2, was referred to our Fetal Medicine Unit for detailed US examination at 20 weeks' gestation after suspicious findings at the routine 20 weeks' scan. The first two pregnancies of this consanguineous couple (half cousins) were uneventful, and their family history was unremarkable for congenital anomalies, except for another half-cousin with an unspecified severe congenital disability.

**Figure 1:**
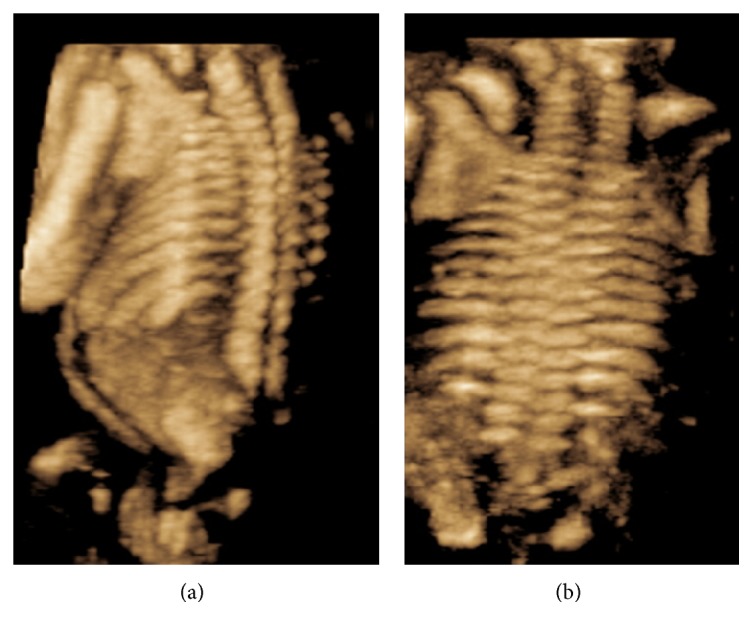
Three-dimensional US scans. (a) 3D rendered image of a lateral view of left upper arm, scapula, chest, and spine. The spine ends abruptly at low lumbar level and appears disconnected from the pelvic bones. (b) 3D rendered coronal image of the fetal neck and trunk. The intervertebral and intercostal spaces appear reduced creating the impression of a compressed fetal trunk.

At the 20-week scan, multiple anomalies were seen in a female fetus: the rump appeared “compressed” with reduced intervertebral and intercostal spaces. As shown in Figure 1, the vertebrae appeared to terminate abruptly at low lumbar level. No clear sacrum was seen and the pelvic bones seemed not to be connected to the spine. An apparent widening of the vertebral canal in cranial direction, above the level of the chest, was also noted.

**Figure 2:**
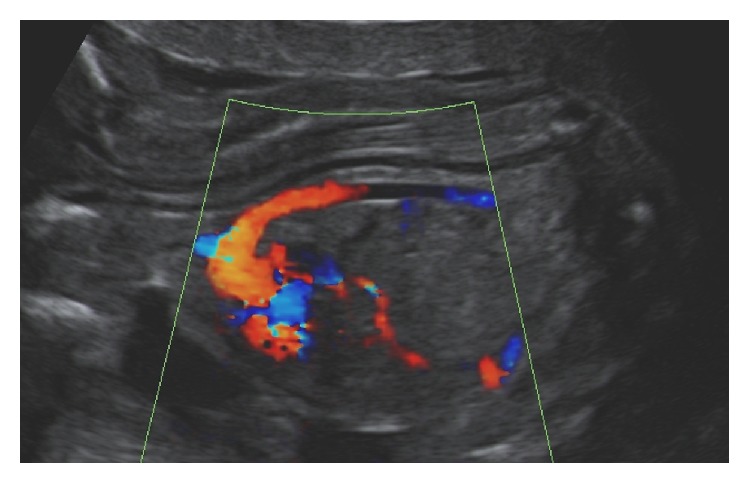
Longitudinal midsagittal view of the upper body of affected fetus. Flow in the aortic arch and descending thoracic aorta is shown by color flow mapping. No flow is visible inside the translucent tubular structure interpreted as the notochord.

Moreover, a nonsegmented tubular translucent structure, positioned in between the spine and the aorta and following the curvature of the fetal spine, was visualized (Figure 2). The structure, with a diameter of 2,7 mm, ran parallel to the vertebral arches exactly where the vertebral bodies, which were not visualized, should have developed. This tubular structure was considered to be the ultrasound appearance of a persistent notochord. In between this structure and the spine a slightly more echogenic space, which tapered caudally, referable to the spinal cord, was observed. The fetus showed also overlapping fingers, bilateral clubfoot, and a single umbilical artery (SUA). The parents declined additional genetic investigations and decided to continue the pregnancy. With advancing gestation, the tubular structure became less evident, the growth was suboptimal (below the 10th percentile), and the amniotic fluid was reduced. Micrognathia and an increased nuchal fold (7,2 mm) were also noted.

At 39 weeks' gestation, the patient delivered vaginally a girl weighing 2990 grams. The Apgar scores were, respectively, 6 and 9, after 1 and 5 minutes. Physical and instrumental examinations of the baby showed limb length discrepancy, anus vestibularis, uterovaginal agenesis, and sacral agenesis with myelomeningocele. CT scans were performed at 3 and 11 months of life and showed two separate ossification centers on both sides of the vertebral bodies (vertebral cleft with two parasagittal ossification centers). Furthermore, recurrent urinary tract infections occurred and a neurogenic bladder was diagnosed. No information is available after her third year of life.

### 2.2. Family B: Case 2

A 21-year-old woman, gravida 2 para 1, with unremarkable medical history, presented at 12 weeks' gestation for a routine first-trimester scan. The ultrasound examination showed a singleton fetus with crown-rump length (CRL) shorter than expected for the gestational age, increased nuchal translucency (3,8 mm), absence of sacrum, contractures of the legs, and bilateral clubfoot. Fetal bladder was dilated and thick walled, while both kidneys were described as echogenic and with dilated renal pelvis. In light of these findings, a chorionic villus sampling was carried out, revealing a normal male karyotype.

At 18 weeks' gestation the mother reported amniotic fluid leakage. Ultrasound examination showed reduced amniotic fluid, dolichocephaly, enlargement of the lateral ventricles, and absence of the cerebellum. The fetal heart appeared enlarged and a ventricular septal defect and moderate pericardial effusion were observed. The bladder was empty and no filling was observed, and the bowel was markedly echogenic. The parents were counseled about the poor prognosis but decided not to terminate the pregnancy.

At 34 weeks the patient delivered a male neonate with an Apgar score of 2 at 5 minutes. The infant died on the same day. The autopsy was declined, while postmortem MRI and CT were performed. These reported the evidence of schizencephaly and agenesis of the corpus callosum. Lumbosacral agenesis was confirmed in addition to myelomeningocele and severe vertebral bodies hypoplasia. The neonate presented also with anal atresia, likely to be associated with an enterovesical fistula.

### 2.3. Family B: Case 3

One year later, the same couple was seen in their third pregnancy. At 11 weeks' gestation, the ultrasound scan showed a fetus with shorter than expected CRL, increased nuchal translucency, a small omphalocele, and unspecified skeletal anomalies with clubfeet. A SUA and mild pericardial effusion were also noticed.

Detailed ultrasound examinations at 13 and 18 weeks revealed sacral agenesis, spina bifida, and abnormal male external genitalia with hypospadia. The parents were informed that the findings suggested a recurrence of the condition in the previous pregnancy. They declined any further invasive investigation. At 21 weeks, the amniotic fluid was reduced, fetal growth was restricted, and the head showed dolichocephaly. Left renal agenesis was also noticed.

At 40 weeks, a male newborn of 2620 grams was delivered vaginally. The infant died on the 3rd day and the parents declined autopsy. Postmortem MRI and X-ray showed a coronal vertical clefting of all vertebral bodies. Furthermore, the reevaluation of the prenatal ultrasound pictures showed the same tubular structure, described in family A and interpreted as a persistent notochord. The structure was detectable at 18 weeks and became gradually less visible with advancing gestation.

## 3. Discussion 

Collectively, the affected fetuses have showed sacral agenesis, abnormal ossification of vertebral bodies, bilateral clubfeet, single umbilical artery, and oligohydramnios from the second trimester onwards. Moreover, an increased nuchal translucency was observed in those presenting early in gestation. As shown in Table 1, most of the prenatal findings in this study are consistent with features of CRS reported in literature, except for the persistent notochord and the association of single umbilical artery and oligohydramnios. The two latter findings, in addition to fusion of the lower limbs, are typical features of sirenomelia, but we did not observe severe anomalies of the lower limbs in our series, a part from clubfeet.

The most striking feature in this case series was the ultrasound visualization of a persistent complete notochordal canal in second-trimester human fetuses. The notochord is a transient embryonic structure that regresses entirely as vertebral bodies form and spinal ossification progresses, while its remnants can be found in the* nuclei pulposi* of the intervertebral discs [6]. Our group has for the first time reported on prenatal visualization a persistent notochord extending along the entire vertebral canal.

All affected fetuses presented normal vertebral arches but abnormal vertebral bodies, consisting of two ossification centers instead of one. In order to understand the meaning of this finding, we should mention that the primitive vertebral body is composed of two chondrification centers, transiently separated by notochord remnants [7] until 7-8 weeks, when a unique ossification core will develop. Studies hypothesized that the persistence of notochord may alter the differentiation of the perinotochordal embryonic cartilage, preventing the physiological ossification of vertebral bodies. This may lead to persistence of two ossification centers instead of one, resulting in a median vertebral cleft [8]. CT scan confirmed the findings of a persistent notochord, located exactly where the vertebral bodies should have developed and associated with median vertebral cleft. A recent report also described the presence of a persistent complete notochord in association with abnormal vertebral bodies in a fetus with hypochondrogenesis caused by COL2A1 mutation [9]. Codsi et al. speculated that, as shown in animal studies, the deficiency of type II collagen may interfere with disappearance of notochord and therefore with normal development of spine. This interpretation further highlights the close correlation between the disappearance of notochord and the physiological development of the spine.

The occurrence of these similar complex anomalies in different families from the same geographical area suggested a common underlying genetic background. The genetic analysis, published by Postma et al. [10], identified a homozygous single base-pair substitution (c.796A>G) in the T gene, in all four affected individuals. All parents were heterozygous for this mutation, and all their unaffected sibs were either heterozygous or wild type. The nucleotide substitution resulted in a mutant protein with reduced activity [10].

Mutations of the brachyury gene (from ancient Greek “short tail”) were first observed in short tailed mice with anomalies of posterior skeleton [11] and in number of presacral vertebrae [12]. For this reason, brachyury gene has been long suspected to play a role in CRS [13]. In addition to these anomalies, T mouse mutants have shown abnormal ossification of vertebral bodies [14], luminal distension of notochord [12], and genitourinary anomalies [15].

Other studies in humans have investigated the involvement of brachyury gene in congenital vertebral malformations phenotypes [16]. Ghebranious et al. [16] concluded that the same T mutation described by Papapetrou et al. [13] (c.1103C>T) substantially increases the risk of sporadically occurred congenital vertebral malformation in humans, but unidentified factors determine their nature, location, and severity. Furthermore, a recent report suggests a link between mutations in the T gene and a Mendelian form of neural tube defects in humans, although with incomplete penetrance [17]. Future studies will further elucidate the role of T gene mutation in the ontogenesis of vertebral anomalies and complex anomalies in the human.

In summary, we have described in detail a human phenotype similar to that observed in T mouse mutants, including the typical ultrasound feature of persistent notochord along the entire vertebral canal, as observed in the second trimester of pregnancy.

This case series suggests that the CRS, considered as the most characteristic embryopathy of diabetic pregnancies, can occur as familiar anomaly in the Currarino syndrome, caused by mutation in the HLXB9 homeobox gene MNX1, and also in this novel syndrome, caused by genetic mutation in the T (brachyury) gene mutation. In light of these findings, in case of prenatal diagnosis of sacral agenesis, we advise to carefully check for abnormal vertebral ossification and rare persistence of the notochord in order to rule the involvement of the T gene.

## Figures and Tables

**Table 1:** Prenatal ultrasound features of caudal regression syndrome: the findings observed in our series are italics.

First trimester	(i) Abnormal appearance of the yolk sac^2^ (ii) *Shorter CRL than expected for the gestational age*^*2*^ (iii) *Increased nuchal translucency*^*2*^ (iv) *Sacral agenesis*^*2*^

Second and third trimester	Spine: (i) *Partial or complete absence of sacrum and sacral vertebrae*^*2*^ (ii) Scoliosis and *kyphosis*^*1*^ (iii) *Abnormal vertebral ossification* (iv) Decreased interspace between femoral heads^1^ Limbs: (i)* Clubfeet*^*1*^ (ii) *Flexion contractures of the lower extremities*^*1*^ (iii) Syndactyly/polydactyly^1^ CNS: (i) *Spina bifida*, meningocele, *or myelomeningocele*^*1*^ (ii) *Hydrocephaly*^*2*^ (iii) Microcephaly, anencephaly, or holoprosencephaly^1^ Face: (i) Pierre Robin syndrome^1^ (ii) Facial clefts^1^ Cardiac: (i) *Ventricular septal defect*^*1*^ (ii) Transposition of great vessels^1^ (iii) Dextrocardia^1^ (iv) Coarctation of the aorta^1^ GU tract: (i) *Renal agenesis*^*1*^ (ii) *Renal dysplasia*^*1*^ (iii) *Hydronephrosis*^*1*^ (iv) Dilated/ectopic ureters^1^ (v) *Ambiguous genitalia*, hypospadias^1^ (vi) Vesical/cloacal exstrophy^1^ (vii) Absent bladder^1^ (viii) *Enlarged and thick-walled bladder* GI tract: (i) *Abdominal wall defect*^*1*^

CNS: central nervous system; GU: genitourinary; GI: gastrointestinal. ^1^Boulas [1]; ^2^Singh et al. [2].

## References

[1] Boulas M. M. (2009). Recognition of caudal regression syndrome. *Advances in Neonatal Care*.

[2] Singh S. K., Singh R. D., Sharma A. (2005). Caudal regression syndrome—case report and review of literature. *Pediatric Surgery International*.

[3] Yeniel A. O., Ergenoglu A. M., Sagol S. (2011). Prenatal diagnosis of caudal regression syndrome without maternal diabetes mellitus. *Journal of the Turkish German Gynecological Association*.

[4] Ogata E. S., MacDonald M. R., Seshia M. M., Mullett M. D. (2005). Carbohydrate homeostasis. *Avery's Neonatology—Pathophysiology and Management of the Newborn*.

[5] Köchling J., Pistor G., Märzhäuser Brands S., Nasir R., Lanksch W. R. (1996). The Currarino syndrome—hereditary transmitted syndrome of anorectal, sacral and presacral anomalies. Case report and review of the literature. *European Journal of Pediatric Surgery*.

[6] Nibu Y., José-Edwards D. S., Di Gregorio A. (2013). From notochord formation to hereditary chordoma: the many roles of brachyury. *BioMed Research International*.

[7] Szpinda M., Baumgart M., Szpinda A., Woźniak A., Mila-Kierzenkowska C. (2013). New patterns of the growing L3 vertebra and its 3 ossification centers in human fetuses—a CT, digital, and statistical study. *Medical Science Monitor Basic Research*.

[8] Taylor J. R. (1972). Persistence of the notochordal canal in vertebrae. *Journal of Anatomy*.

[9] Codsi E., Brost B. C., Faksh A., Volk A. K., Borowski K. S. (2015). Persistent notochord in a fetus with COL2A1 mutation. *Case Reports in Obstetrics and Gynecology*.

[10] Postma A. V., Alders M., Sylva M. (2014). Mutations in the T (brachyury) gene cause a novel syndrome consisting of sacral agenesis, abnormal ossification of the vertebral bodies and a persistent notochordal canal. *Journal of Medical Genetics*.

[11] Chesley P., Dunn L. C. (1936). The inheritance of taillessness (anury) in the house mouse. *Genetics*.

[12] Gruneberg H. (1958). Genetical studies on the skeleton of the mouse XXII. The development of brachyury and anury. *Journal of Embryology and Experimental Morphology*.

[13] Papapetrou C., Drummond F., Reardon W., Winter R., Spitz L., Edwards Y. H. (1999). A genetic study of the human T gene and its exclusion as a major candidate gene for sacral agenesis with anorectal atresia. *Journal of Medical Genetics*.

[14] Gluecksohn-Schoenheimer S. (1938). The development of two tailless mutants in the house mouse. *Genetics*.

[15] Park C.-H. T., Pruitt J. H., Bennett D. (1989). A mouse model for neural tube defects: the *Curtailed* (*T*^*c*^) mutation produces spina bifida occulta in *T*^*c*^/ + animals and spina bifida with meningomyelocele in *T*^*c*^/*t*. *Teratology*.

[16] Ghebranious N., Blank R. D., Raggio C. L. (2008). A missense *T* (*Brachyury*) mutation contributes to vertebral malformations. *Journal of Bone and Mineral Research*.

[17] Shaheen R., Alshail E., Alaqeel A., Ansari S., Hindieh F., Alkuraya F. S. (2015). T (brachyury) is linked to a Mendelian form of neural tube defects in humans. *Human Genetics*.

